# Beyond the Ocular Surface: Nasal Sensory Input as a Driver of Reflex Lacrimation in Dogs

**DOI:** 10.1111/vop.70108

**Published:** 2025-11-07

**Authors:** Shelley W. Cochran, Nick J. Millichamp, Lionel Sebbag

**Affiliations:** ^1^ Eye Care for Animals, Thrive Pet Healthcare Houston Texas USA; ^2^ Koret School of Veterinary Medicine The Hebrew University of Jerusalem Rehovot Israel

**Keywords:** aqueous tear production, brachycephalic, dry eye disease, lacrimation, nasolacrimal reflex, reflex tearing

## Abstract

**Objective:**

To evaluate the effect of nasal mucosal anesthesia on aqueous tear secretion in dogs and to compare responses between brachycephalic and non‐brachycephalic breeds.

**Animal Studied:**

Twenty healthy dogs (10 Australian Shepherds, 10 Boston Terriers).

**Procedures:**

All dogs received 0.5 mL of 10% lidocaine or saline into one randomly selected nostril. The alternate solution was administered in the same nostril 2 weeks later. Schirmer tear test‐1 (STT‐1) was performed bilaterally before and 15 min after nasal administration. Tear strip wetting was recorded every 10 s for 60 s; the initial uptake phase (0–10 s) reflected uptake of pre‐existing tears, while the active secretion phase (10–60 s) represented reflex tearing. Statistical comparisons included paired t‐tests and linear mixed‐effects models.

**Results:**

In non‐brachycephalic dogs, lidocaine significantly reduced STT‐1 values in the treated side by 11.5% (20.0–17.7 mm, *p* = 0.045) and did not cause a significant change in the contralateral side (21.7–20.1 mm, −7.4%, *p* = 0.280). Reflex tear slope decreased by 21.7% (0.23–0.18 mm/s, *p* = 0.004), while the initial phase slope remained unchanged (0.84–0.88 mm/s, *p* = 0.653). In brachycephalic dogs, lidocaine had no significant effect in either eye or tear phase (*p* ≥ 0.132). Saline caused mild, non‐significant increases in STT‐1 across all groups (+0.4% to 8.4%, *p* ≥ 0.116). Mixed‐effects analysis identified skull type as the only significant predictor of treatment response (*p* = 0.047).

**Conclusions:**

Nasal mucosal anesthesia reduced reflex tear production in dogs, particularly in non‐brachycephalic breeds. These results confirm the presence of a functional nasolacrimal reflex in dogs and suggest diminished nasal sensory input in brachycephalic breeds.

## 1 Introduction

Aqueous tear production is regulated by both basal secretion and reflex pathways, the latter being triggered by sensory input from the ocular surface and nasal mucosa [1, 2]. In dogs, decreased corneal sensitivity due to endocrinopathies, ocular pathology, or brachycephalic conformation is a well‐known contributor to reduced tear production and clinical signs consistent with keratoconjunctivitis sicca [3, 4, 5]. In contrast, the contribution of nasal sensory input to tear production in dogs remains poorly understood.

Nasal mucosal stimulation activates the nasolacrimal reflex (NLR), with afferent signals transmitted via branches of the trigeminal nerve and efferent activation of the lacrimal gland through parasympathetic pathways [1, 2, 3]. In humans, the NLR can account for up to one‐third of reflex tearing and is attenuated in dry eye disease [1, 2, 6], where its diagnostic and therapeutic relevance has spurred the development of neurostimulatory devices and intranasal pharmacologic agents [7, 8, 9, 10, 11, 12].

Despite growing interest in comparative ophthalmology and tear physiology, the NLR has received little attention in dogs. A recent canine study showed that chemical stimulation near the nostrils increased aqueous tear production by 18%, suggesting a functional NLR in dogs [3]. Similar findings have been reported in cats with metaherpetic disease and neurogenic dry eye [13, 14]. However, no veterinary study has directly quantified the impact of nasal sensory input on lacrimation. This question is particularly relevant in brachycephalic breeds, which often have altered nasal anatomy (e.g., stenotic nares, turbinate crowding, redundant mucosa) that may impair sensory input [15, 16]. These breeds also show decreased corneal sensitivity, suggesting a potential generalized trigeminal hypoesthesia.

This study aimed to evaluate the functional role of the NLR in dogs by selectively anesthetizing the nasal mucosa and measuring temporal changes in tear production. We hypothesized that nasal anesthesia would suppress aqueous tear secretion, with differences based on cephalic conformation.

## 2 Materials and Methods

This prospective, crossover study included 20 healthy, client‐owned dogs. The protocol was approved by the Thrive Pet Healthcare research committee and written informed consent was obtained from all owners.

### 2.1 Animals

The cohort consisted of 10 Australian Shepherds (non‐brachycephalic) and 10 Boston Terriers (brachycephalic). The Australian Shepherds consisted of 7 females (2 spayed, 5 intact) and 3 males (1 intact, 2 neutered), aged 6.3 ± 4.7 years (1.5–13 years) and weighing 19.8 ± 3.9 kg (15–27 kg). The Boston Terriers included 9 intact females and 1 intact male, aged 3.3 ± 2.0 years (1–6 years) and weighing 6.7 ± 1.0 kg (5.2–8.2 kg). All dogs underwent a complete ophthalmic examination by a board‐certified veterinary ophthalmologist (NJM), including slit‐lamp biomicroscopy (SL‐19; Kowa), indirect ophthalmoscopy (Heine Omega 500), rebound tonometry (TonoVet, Icare), and baseline Schirmer tear test‐1 (STT‐1) confirming tear production > 15 mm/min. Corneal tactile sensation (CTS) was assessed using a Cochet‐Bonnet aesthesiometer (Luneau Ophtalmologie) to verify intact afferent innervation, as previously described [3]. Craniofacial ratio (CFR; muzzle length/skull length) was measured using a ruler, with a value ≤ 0.49 defining brachycephaly [15].

### 2.2 Treatment Protocol

All exams and procedures were performed in the same indoor exam room during afternoon hours (1 PM–6 PM), with temperature and humidity recorded on each testing day. Each dog served as its own control, receiving both intranasal 10% lidocaine (Stokes Pharmacy) and saline (Eye Relief; Bausch and Lomb) on separate occasions, with a 2‐week washout period. Dogs were gently restrained in a sitting position with their head tilted upward, and 0.5 mL of the test solution was administered into one randomly selected nostril using a MILA VetJet. The contralateral nostril was left untreated.

STT‐1 was performed in both eyes at baseline and 15 min after intranasal administration, a timing selected to allow adequate tear film replenishment [17]. Dye‐free Schirmer strips (Merck Animal Health) were placed in the ventrolateral conjunctival fornix for 60 s. Wetting length was recorded every 10 s. The 0–10 s interval represented the *initial uptake* phase, reflecting absorption of pre‐existing tear fluid from the lacrimal pool. The 10–60 s interval represented the *active secretion* phase (reflex tearing), driven by reflex tear production in response to strip insertion and sensory input [18].

### 2.3 Outcomes Measured

The primary outcome was STT‐1 at 60 s (mm/min). Secondary outcomes included the slopes of lacrimation kinetics during the initial uptake (0–10 s) and active secretion (10–60 s) phases, as well as the influence of skull type, CFR, sex, age, and body weight.

### 2.4 Data Analysis

An a priori sample size calculation was performed using published STT‐1 values in brachycephalic and non‐brachycephalic dogs [3]. Assuming a 25%–30% reduction in tear production with attenuation of the nasolacrimal reflex (based on human studies) [1, 2, 6], we estimated that 5–6 brachycephalic dogs and 8–10 non‐brachycephalic dogs would be required to achieve > 80% power at a significance level (α) of 0.05. Thus, the total sample of 20 dogs provided adequate statistical power for this study. Normality of data was assessed with the Shapiro–Wilk test. Paired *t*‐tests compared pre‐ and post‐treatment values. A linear mixed‐effects model evaluated the influence of skull type, eye laterality, sex, and body weight on tear production. Analyses were performed using SigmaPlot v15.0 (Systat Inc., USA), with significance set at *p* < 0.05.

## 3 Results

**FIGURE 1:**
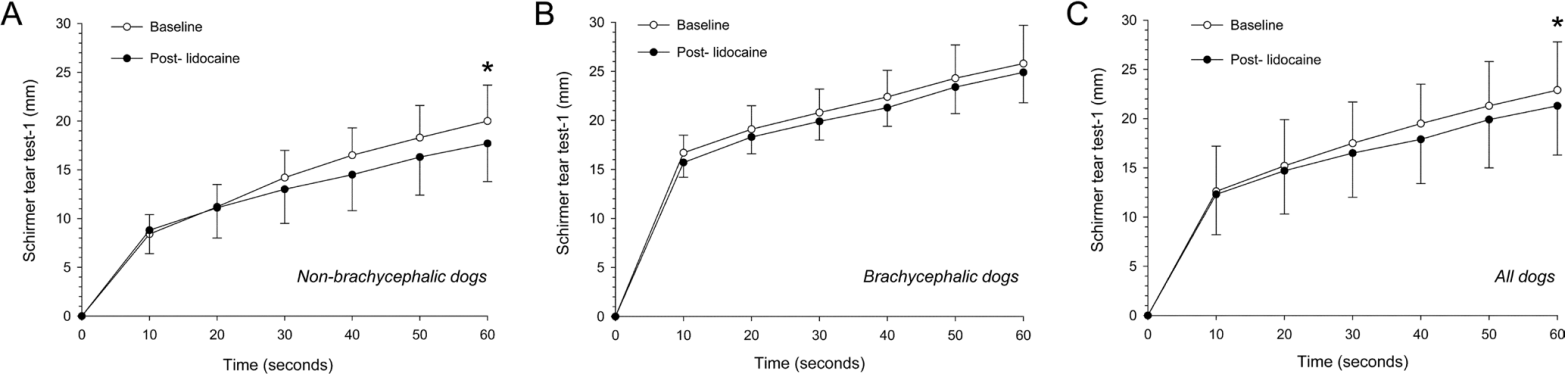
Scatter plots depicting mean ± standard deviation Schirmer tear test‐1 values in the ipsilateral eye of 20 dogs before (white circles) and 15 min after (black circles) intranasal lidocaine administration in one nostril. Data are plotted every 10 s for 60 s in non‐brachycephalic dogs (A, *n* = 10), brachycephalic dogs (B, *n* = 10), and all dogs combined (C, *n* = 20). The 0–10 s interval represents the initial uptake phase (basal tearing), and the 10–60 s interval represents the active secretion phase (reflex tearing).

Environmental conditions ranged from 21°C to 23°C and 46%–51% humidity. Mean ± standard deviation (range) for CTS and CFR were 3.1 ± 0.7 cm (2–4 cm) and 0.71 ± 0.06 (0.62–0.84) in non‐brachycephalic dogs, and 2.4 ± 0.6 cm (1–3 cm) and 0.19 ± 0.03 (0.15–0.25) in brachycephalic dogs. Data are summarized in Tables 1 and 2 and Figure 1.

**TABLE 1:** Mean ± SD (range) of Schirmer tear test‐1 values at 60 s in 20 dogs (10 non‐brachycephalic, 10 brachycephalic) before and 15 min after intranasal lidocaine or saline administration. Each dog received both treatments in a randomized crossover design with a 2‐week washout. Tear production was measured in the ipsilateral and contralateral eyes. Changes from baseline were assessed using paired *t*‐tests.

			Non‐brachycephalic	Brachycephalic	All dogs
Lidocaine	Ipsilateral	Baseline	20.0 ± 3.7 (15–25)	25.8 ± 4.3 (20–35)	22.9 ± 4.9 (15–35)
		Post‐	17.7 ± 3.9 (9–22)	24.9 ± 3.1 (20–31)	21.3 ± 5.0 (9–31)
		% change	–11.5%	–3.5%	–7.0%
		*p* value	**0.045**	0.262	**0.019**
	Contralateral	Baseline	21.7 ± 4.3 (15–30)	24.8 ± 2.9 (21–30)	23.3 ± 3.9 (15–30)
		Post‐	20.1 ± 4.6 (12–27)	25.4 ± 3.8 (20–30)	22.8 ± 4.9 (12–30)
		% change	–7.4%	+2.4%	–2.1%
		*p* value	0.280	0.568	0.574
Saline	Ipsilateral	Baseline	18.2 ± 2.9 (15–23)	24.2 ± 3.2 (18–28)	21.2 ± 4.3 (15± –28)
		Post‐	18.8 ± 4.1 (11–27)	24.3 ± 5.1 (18–34)	21.6 ± 5.3 (11–34)
		% change	+3.3%	+0.4%	+1.9%
		*p* value	0.572	0.936	0.657
	Contralateral	Baseline	20.2 ± 3.7 (15–25)	24.8 ± 3.8 (18–30)	23.0 ± 4.5 (15–31)
		Post‐	21.9 ± 3.4 (15–26)	25.7 ± 3.4 (21–31)	23.4 ± 3.8 (15–30)
		% change	+8.4%	+3.5%	+1.7%
		*p* value	0.116	0.422	0.607

*Note:* Values in bold are significant (*p* < 0.05).

**TABLE 2:** Mean ± SD (range) slopes of basal tearing (0–10 s) and reflex tearing (10–60 s) in 20 dogs (10 non‐brachycephalic, 10 brachycephalic) before and 15 min after intranasal lidocaine or saline administration. Each dog received both treatments in a randomized crossover design with a 2‐week washout. Tear production was measured in the ipsilateral and contralateral eyes. Changes from baseline were assessed using paired *t*‐tests.

			Non‐brachycephalic		Brachycephalic		All dogs	
			Basal (0–10 s)	Reflex (10–60 s)	Basal (0–10 s)	Reflex (10–60 s)	Basal (0–10 s)	Reflex (10–60 s)
Lidocaine	Ipsilateral	Baseline	0.84 ± 0.20 (0.5–1.2)	0.23 ± 0.05 (0.16–0.3)	1.67 ± 0.18 (1.5–2.0)	0.18 ± 0.07 (0.1–0.36)	1.26 ± 0.46 (0.5–2.0)	0.21 ± 0.06 (0.1–0.36)
		Post‐	0.88 ± 0.24 (0.6–1.3)	0.18 ± 0.06 (0.06–0.28)	1.57 ± 0.15 (1.4–1.9)	0.18 ± 0.05 (0.12–0.32)	1.23 ± 0.41 (0.6–1.9)	0.18 ± 0.06 (0.06–0.32)
		% change	+4.8%	–21.7%	–6.0%	0%	–2.4%	–14.3%
		*p* value	0.653	**0.004**	0.186	0.780	0.600	**0.018**
	Contralateral	Baseline	0.86 ± 0.20 (0.6–1.2)	0.26 ± 0.06 (0.18–0.36)	1.65 ± 0.16 (1.5–2.0)	0.17 ± 0.03 (0.12–0.20)	1.26 ± 0.44 (0.6–2.0)	0.21 ± 0.07 (0.12–0.36)
		Post‐	0.84 ± 0.32 (0.5–1.4)	0.23 ± 0.06 (0.12–0.32)	1.64 ± 0.3 (1.2–2.0)	0.18 ± 0.03 (0.14–0.22)	1.24 ± 0.51 (0.5–2.0)	0.21 ± 0.05 (0.12–0.32)
		% change	–2.3%	–11.5%	–0.6%	+5.9%	–1.6%	0%
		*p* value	0.861	0.153	0.906	0.132	0.826	0.524
Saline	Ipsilateral	Baseline	0.81 ± 0.17 (0.5–1.0)	0.20 ± 0.05 (0.12–0.28)	1.58 ± 0.23 (1.2–1.9)	0.17 ± 0.02 (0.12–0.20)	1.2 ± 0.44 (0.5–1.9)	0.19 ± 0.04 (0.12–0.28)
		Post‐	0.79 ± 0.17 (0.5–1.0)	0.22 ± 0.06 (0.12–0.34)	1.6 ± 0.44 (1.1–2.5)	0.17 ± 0.02 (0.14–0.20)	1.2 ± 0.53 (0.5–2.5)	0.19 ± 0.05 (0.12–0.34)
		% change	–2.5%	+10%	+1.3%	0%	0%	0%
		*p* value	0.716	0.405	0.859	0.726	1.000	0.472
	Contralateral	Baseline	0.99 ± 0.3 (0.6–1.5)	0.23 ± 0.06 (0.18–0.34)	1.66 ± 0.36 (1.0–2.2)	0.18 ± 0.02 (0.14–0.22)	1.28 ± 0.51 (0.6–2.2)	0.2 ± 0.05 (0.14–0.34)
		Post‐	1.04 ± 0.4 (0.5–2.0)	0.21 ± 0.06 (0.1–0.32)	1.65 ± 0.28 (1.1–2.0)	0.17 ± 0.03 (0.12–0.2)	1.4 ± 0.42 (0.5–2.0)	0.19 ± 0.05 (0.1–0.32)
		% change	+5.1%	–8.7%	–0.6%	–5.6%	+9.4%	–5%
		*p* value	0.265	0.458	0.929	0.168	0.126	0.173

*Note:* Values in bold are significant (*p* < 0.05).

### 3.1 Lidocaine Treatment

In non‐brachycephalic dogs, lidocaine significantly reduced mean STT‐1 values in the treated side by 11.5% (20.0 to 17.7 mm, *p* = 0.045) and reduced values in the contralateral side by 7.4% (21.7 to 20.1 mm), although this change was not statistically significant (*p* = 0.280). Reflex tear slope significantly declined by 21.7% (0.23 to 0.18 mm/s, *p* = 0.004), with no significant change in the initial phase slope (0.84 to 0.88 mm/s, *p* = 0.653).

In brachycephalic dogs, lidocaine did not cause a significant change in the treated side (25.8 to 24.9 mm, −3.5%, *p* = 0.262) and contralateral side (24.8 to 25.4 mm, +2.4%, *p* = 0.568). Neither the reflex (0.18 to 0.18 mm/s) nor initial phase (1.67 to 1.57 mm/s) slopes was significantly altered (*p* ≥ 0.132).

Across all 20 dogs, lidocaine significantly reduced tear production in the treated side by 7.0% (22.9 to 21.3 mm, *p* = 0.019), and reflex tear slope by 14.3% (0.21 to 0.18 mm/s, *p* = 0.018), with no change in the initial phase slope (1.26 to 1.23 mm/s, *p* = 0.600). Tear production in the contralateral side decreased by 2.1% but did not reach significance (*p* = 0.574). Skull type was the only significant predictor of tear suppression (*p* = 0.047, linear mixed model).

### 3.2 Saline Treatment

Saline administration resulted in mild, non‐significant increases (*p* ≥ 0.116) in tear production across all groups (+0.4% to 8.4%) and did not affect reflex or initial phase slopes (*p* ≥ 0.126).

## 4 Discussion

This study provides novel experimental evidence that nasal sensory input contributes meaningfully to aqueous tear production in dogs. The administration of intranasal lidocaine significantly reduced reflex tearing as measured by the active secretion slope of the STT‐1, particularly in non‐brachycephalic dogs, confirming the functional presence of the nasolacrimal reflex in this species.

The magnitude and specificity of the reflex suppression observed in this study closely mirror findings in human medicine [1, 2]. In people, nasal stimulation activates afferent fibers of the anterior ethmoidal and maxillary branches of the trigeminal nerve, which in turn engage the superior salivatory nucleus and parasympathetic efferents to the lacrimal gland [1]. The NLR serves as the basis for emerging intranasal devices and pharmacologic agents for dry eye therapy in human patients, such as the TrueTear device or varenicline nasal spray (Tyrvaya) [7, 8, 11, 12, 19]. The canine NLR, although less studied, appears to share key neuroanatomical and physiological characteristics with the human model, raising the possibility of translational relevance for both diagnostic and therapeutic applications.

The decision to analyze STT kinetics using a two‐phase model was informed by prior studies suggesting that tear strip wetting is not linear over time [18, 20, 21]. In particular, Williams (2005) demonstrated an initial rapid rise in wetting—attributed to uptake from the pre‐existing tear lake—followed by a slower, steady‐state phase reflecting ongoing tear secretion [18]. While not formally labeled as “basal” and “reflex” phases in earlier studies, this temporal distinction has provided valuable insights into tear dynamics in dogs with both normal and abnormal lacrimal function. In the present study, we adopted a similar time‐based division (0–10 and 10–60 s) to differentiate between passive uptake and active secretion, enabling a more refined assessment of reflex tearing in response to nasal sensory input. With this in mind, we observed that lidocaine‐induced suppression was largely confined to the 10–60 s interval of the STT‐1, which we defined as the active secretion phase (reflex tearing). The lack of significant changes in initial uptake suggests that lidocaine did not produce a generalized reduction in lacrimal gland output or induce sedation that would affect basal tear production. Instead, the data support a targeted interruption of reflex pathways. Although systemic absorption of lidocaine likely occurred given the vascularity of the nasal mucosa, the small administered volume (0.5 mL), absence of contralateral effects, and evidence that intravenous lidocaine does not decrease tear production in dogs [22], all suggest our findings reflect a local nasal effect rather than a systemic pharmacologic action.

Brachycephalic dogs showed minimal to no suppression of tearing following lidocaine administration. This observation likely reflects the unique craniofacial anatomy of brachycephalic breeds, including shortened nasal passages, narrowed nares, redundant mucosal folds, and turbinate crowding [15]. These structural differences may diminish airflow, reduce mucosal surface area, and ultimately attenuate sensory input from the nasal cavity. Additionally, prior studies have demonstrated reduced corneal sensitivity in brachycephalic dogs [3], raising the possibility of generalized trigeminal hypoesthesia. Thus, the diminished NLR activity observed in these dogs may be due to both anatomical and neurophysiological alterations.

The minimal change observed in the contralateral side following lidocaine treatment suggests that the canine NLR operates predominantly through an ipsilateral reflex arc, consistent with findings in human studies [1, 2]. While slight reductions were noted in the untreated side of non‐brachycephalic dogs, these changes did not reach statistical significance. Future work incorporating bilateral treatments or electrophysiological assessments may help clarify the degree of cross‐communication between nasal and lacrimal pathways. The saline control arm provided an important comparator, confirming that the reductions observed with lidocaine were not attributable to fluid volume, administration technique, or temperature effects. Interestingly, tear production occasionally increased following saline administration, likely due to mechanical stimulation or mild irritation of the nasal mucosa. These findings further emphasize the specificity of the anesthetic effect in suppressing the NLR.

From a clinical perspective, reflex tearing is a critical protective mechanism that defends the ocular surface against desiccation, allergens, and pathogens. Impairment of this mechanism, whether due to nasal structural abnormalities or sensory neuropathy, could predispose dogs to ocular surface disease. In brachycephalic dogs, where tear film instability and ocular surface pathology are already prevalent, reduced NLR function may represent an underrecognized contributor. Enhancing NLR activity through neurostimulation or other interventions may offer therapeutic benefits in these patients [10].

It is worth noting that this study likely underestimates the full contribution of the NLR [1]. Only one side of the nasal cavity was anesthetized, leaving the contralateral side potentially active and capable of triggering reflex tearing. Bilateral anesthesia may result in a more substantial suppression of tearing and should be explored in future studies. Unilateral treatment was selected here for practical reasons: initial pilot attempts at bilateral treatment led to poor compliance, and the crossover design enabled each dog to serve as its own control. Of note, the magnitude of the STT‐1 changes was relatively small and unlikely to be clinically meaningful, as unilateral nasal anesthesia did not result in observable ocular surface dryness.

This study has several limitations. The sample size, while sufficient to detect major effects, may have limited our ability to identify more subtle differences, particularly in brachycephalic dogs. Additionally, although lidocaine is primarily known for its anesthetic properties, it may exert other effects on mucosal vasculature or epithelial permeability that could theoretically influence tear production. However, the use of a saline control and the selective suppression of reflex tearing make this unlikely to be the dominant mechanism. We used lidocaine 10% rather than ophthalmic anesthetics such as proparacaine or tetracaine (0.5%–1%), since only a small intranasal volume (0.5 mL) could be administered for compliance, and a higher concentration was needed to ensure effective nasal mucosal anesthesia.

In conclusion, unilateral nasal mucosal anesthesia with lidocaine significantly reduced reflex tear production in dogs without affecting initial tear output. This effect was most evident in non‐brachycephalic breeds and supports the existence of a functional nasolacrimal reflex. In contrast, brachycephalic dogs showed minimal response, suggesting reduced reflex tearing capacity likely due to anatomical and neurophysiological alterations. These findings help explain the predisposition of brachycephalic breeds to dry eye and support further exploration of the nasolacrimal reflex as a diagnostic and therapeutic target in veterinary ophthalmology.

## Disclosure

Artificial intelligence statement: The authors have not used AI to generate any part of the manuscript.

## Ethics Statement

The protocol was approved by the Thrive Pet Healthcare research committee and written informed consent was obtained from all owners.

## Conflicts of Interest

The authors declare no conflicts of interest.

## Data Availability

The data that support the findings of this study are available from the corresponding author upon reasonable request.
